# Identification of Long Noncoding RNAs Deregulated in Papillary Thyroid Cancer and Correlated with BRAF^V600E^ Mutation by Bioinformatics Integrative Analysis

**DOI:** 10.1038/s41598-017-01957-0

**Published:** 2017-05-10

**Authors:** Lucas Goedert, Jessica Rodrigues Plaça, Cesar Seigi Fuziwara, Maiaro Cabral Rosa Machado, Desirée Rodrigues Plaça, Palloma Porto Almeida, Talita Perez Sanches, Jair Figueredo dos Santos, Amanda Cristina Corveloni, Illy Enne Gomes Pereira, Marcela Motta de Castro, Edna Teruko Kimura, Wilson Araújo Silva Jr., Enilza Maria Espreafico

**Affiliations:** 1 Department of Cell and Molecular Biology, Faculty of Medicine of Ribeirão Preto, University of São Paulo, Ribeirão Preto, São Paulo, Brazil; 2 National Institute of Science and Technology in Stem Cell and Cell Therapy and Center for Cell-Based Therapy, Ribeirão Preto, São Paulo, Brazil; 3 Clinical Oncology, Stem Cell and Cell Therapy Program, Ribeirão Preto Medical School, Ribeirão Preto, São Paulo, Brazil; 4 Department of Cell and Developmental Biology, Institute of Biomedical Sciences, University of São Paulo, São Paulo, São Paulo, Brazil; 5 Biotechnology Program, Federal University of São Paulo, São José dos Campos, São Paulo, Brazil; 6 Biotechnology Program, Federal University of Bahia, Vitória da Conquista, Bahia, Brazil; 7 Biomedicine Program, Federal University of Pernambuco, Recife, Pernambuco Brazil; 8 Biomedicine Program, State University of Londrina, Londrina, Paraná, Brazil; 9 Department of Genetics, Ribeirão Preto Medical School, and Center for Integrative System Biology – CISBi-NAP/USP, University of São Paulo, Ribeirão Preto, São Paulo, Brazil

## Abstract

Papillary Thyroid Cancer (PTC) is an endocrine malignancy in which BRAF^V600E^ oncogenic mutation induces the most aggressive phenotype. In this way, considering that lncRNAs are arising as key players in oncogenesis, it is of high interest the identification of BRAF^V600E^-associated long noncoding RNAs, which can provide possible candidates for secondary mechanisms of BRAF-induced malignancy in PTC. In this study, we identified differentially expressed lncRNAs correlated with BRAF^V600E^ in PTC and, also, extended the cohort of paired normal and PTC samples to more accurately identify differentially expressed lncRNAs between these conditions. Indirectly validated targets of the differentially expressed lncRNAs in PTC compared to matched normal samples demonstrated an involvement in surface receptors responsible for signal transduction and cell adhesion, as well as, regulation of cell death, proliferation and apoptosis. Targets of BRAF^V600E^-correlated lncRNAs are mainly involved in calcium signaling pathway, ECM-receptor interaction and MAPK pathway. In summary, our study provides candidate lncRNAs that can be either used for future studies related to diagnosis/prognosis or as targets for PTC management.

## Introduction

Thyroid cancer is the endocrine malignancy^1^ that, although stable until the 1990s, has progressively and greatly increased thereafter^2, 3^. The vast majority of the thyroid cancers originate from the follicular cell epithelium^1^, which includes papillary thyroid carcinoma (PTC) that accounts for approximately 80% of all thyroid malignancies^4^.

Thyroid oncogenesis is still under investigation, however a high frequency (70%) of activating mutations in components of the mitogen-activated protein kinase (MAPK) pathway was reported, such as BRAF^V600E^ ^5, 6^ and HRAS/NRAS/KRAS point mutations^7, 8^. Also, fusions involving the RET^9^ and NTRK1 tyrosine kinases^10^ were described to promote thyroid cancers. More recently, the set of known PTC driver alterations was extended to include EIF1AX, PPM1D, and CHEK2^7^.

Additionally of being the most frequent mutation in many types of cancers including PTC^7, 11^, BRAF^V600E^ confers poorer prognosis compared to other oncogenes. There is a growing number of evidence demonstrating that BRAF^V600E^ correlates with metastasis, cancer recurrence^12^ and higher mortality in PTC^13^. BRAF^V600E^-expressing cells have a diversity of malignant characteristics, including increased DNA synthesis, dedifferentiation, and chromosomal instability^14^. Also, BRAF^V600E^ stimulates more actively MEK-dependent invasion than the expression of RET/PTC oncoprotein through the expression of matrix metalloproteinases (e.g. MMP-3, MMP-9 and MMP-13), which, in part, can explain the more aggressive BRAF^V600E^-induced phenotype^15^.

Similarly to melanoma^16^, BRAF mutation occurs at early stages of PTC development^11, 17^. Besides all BRAF oncogenic activities, its single exacerbated stimulation of the MAPK pathway is not sufficient to sustain malignant transformation, resulting in induced senescence^18^ that confers a barrier to tumor progression^19^. To bypass BRAF-induced senescence, cells may suffer a second event that allows malignant transformation, as possibly the epigenetic silencing of tumor suppressors DAPK, TIMP3^20^, SLC5A8^20, 21^ and hMLH1^22^ and other BRAF-induced mechanisms that remain to be discovered^11^. In thyroid cancers, Thyroid-stimulating Hormone (TSH) is more involved in overcoming senescence; while BRAF overexpression suppresses thyroid hormone biosynthesis and leads to elevated TSH levels *in vivo* ^14^; it was shown that TSH signaling inhibits BRAF^V600E^-induced senescence through DUSP6^23^.

Recently, BRAF^V600E^-associated mRNA signature was determined in a mouse model and human samples^24^, which identified new genes not previously reported as related to BRAF mutation in thyroid cancer (e.g. MMD, ITPR3, AACS, LAD1, PVRL3, ALDH3B1, and RASA1) that will provide further support for future research on BRAF-induced PTC^24^. However, this analysis did not evaluate the expression of long noncoding RNAs (lncRNAs), which are progressively shown to be of fundamental importance in other types of cancer^25, 26^. Such analysis is necessary for the identification of BRAF^V600E^-correlated long noncoding RNAs.

LncRNAs are RNAs longer than 200 nucleotides that have no coding potential^27^ and are involved in several processes, such as gene expression regulation through chromatin modulation^28, 29^, epigenetic control^30^, association with translational apparatus^31^, improving other mRNA stability^32^, serving as a scaffold for protein^33^, acting as decoys for miRNAs^34^, altering protein turnover^35^, among others.

To date, as per the authors’ knowledge, only a few published studies identified differentially expressed (DE) lncRNAs between normal (N) and tumoral thyroid (T) in a limited set of paired samples^36, 37^. Although these findings laid the foundation for further investigation of lncRNAs related to PTC^36^, they need to be confirmed in a more numerous group of patients.

In this study we confirmed previously reported lncRNAs and determined new DE lncRNAs in PTC in a larger set of samples and also identified BRAF^V600E^-correlated lncRNAs, providing possible candidates that can constitute secondary mechanisms of BRAF- induced malignancy in PTC.

## Results

### Comparative analyses identified lncRNAs deregulated in PTC and correlated with BRAF^V600E^ mutation

**Figure 1:**
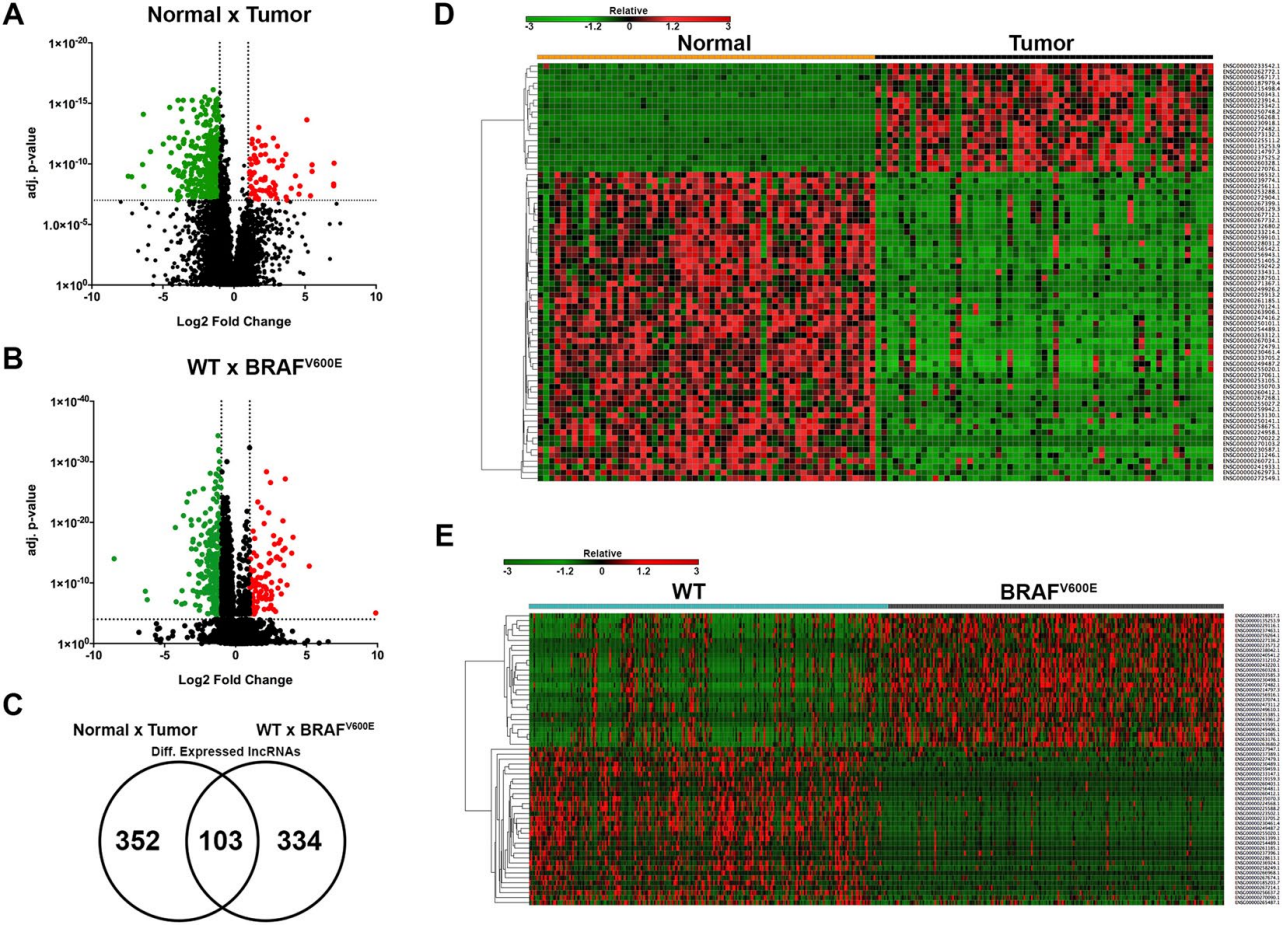
Differentially expressed lncRNAs between N × T and WT × BRAF^V600E^ were identified. (**A**) Volcano plot of DE lncRNAs between N × T (log2 fold change > 1 or < −1; adj. p-value < 1 × 10^−7^). (**B**) Volcano plot of DE lncRNAs between WT × BRAF^V600E^ (log2 fold change > 1 or < −1; adj. p-value < 1 × 10^−4^). (**C**) Venn Diagram of common DE lncRNAs between N × T and WT × BRAF^V600E^. (**D**) Heatmap* of DE lncRNAs between N × T (log2 fold change > 3 or < −3; adj. p-value < 1 × 10^−7^). (**E**) Heatmap* of DE lncRNAs between WT × BRAF^V600E^ (log2 fold change > 2.5 or < −2.5; adj. p-value < 1 × 10^−4^). *For hierarchical clustering, one minus Spearman rank correlation was performed.

Comparative analysis between 59 pairs of matched normal and papillary thyroid cancer samples identified 455 differentially expressed lncRNAs (log2 fold change > 1 or < −1; adj. p-value < 1 × 10^−7^; Fig. 1A), being 71 lncRNAs upregulated and 384 lncRNAs downregulated in PTC (Supplemental Table S1). The same samples presented a total of 2016 mRNAs (log2 fold change > 1 or <−1; adj. p-value < 0.05; Supplemental Table S2) and 186 microRNAs (log2 fold change > 1 or < −1; adj. p-value < 0.05; Supplemental Table S3) differentially expressed.

Differential expression analyses were also performed to identify BRAF^V600E^-correlated lncRNAs. The comparison between BRAF wild type (WT) patients (n = 242) and BRAF^V600E^ patients (n = 226), determined 437 differentially expressed lncRNAs (log2 fold change > 1 or < −1; adj. p-value 1X10^−4^; Fig. 1B), being 117 upregulated and 320 downregulated (Supplemental Table S4). The same comparison found a total of 924 mRNAs (log2 fold change > 1 or < −1; adj. p-value < 0.05; Supplemental Table S5) and 94 microRNAs (log2 fold change > 1 or < −1; adj. p-value < 0.05; Supplemental Table S6) differentially expressed. A total of 103 lncRNAs was differentially expressed in both analyses [(Normal × Tumor and WT × BRAF^V600E^), (Fig. 1C, Table 1 and Supplemental Table S7)].

**Table 1:** Top 5 upregulated and 5 downregulated common DE lncRNAs between Normal × Tumor and WT × BRAF^V600E^.

Ensembl	log2 FC	Adj. p-value	log2 FC	Adj. p-value
	N × T	N × T	WT × BRAF^V600E^	WT × BRAF^V600E^
*ENSG00000214797.3*	7.01	6.39E-09	5.19	4.92E-12
*ENSG00000273132.1*	5.49	4.17E-10	1.69	3.70E-12
*ENSG00000230918.1*	5.49	1.17E-10	1.36	3.89E-05
*ENSG00000260328.1*	5.39	4.27E-08	4.04	2.26E-16
*ENSG00000256268.1*	5.12	2.27E-14	1.00	4.52E-13
*ENSG00000261185.1*	−6.42	1.13E-10	−2.81	3.55E-06
*ENSG00000254489.1*	−6.37	7.98E-15	−3.27	8.33E-11
*ENSG00000260412.1*	−6.33	7.11E-09	−3.77	2.59E-08
*ENSG00000235070.3*	−4.97	2.28E-12	−3.01	1.10E-15
*ENSG00000247416.2*	−4.55	6.89E-13	−2.40	1.09E-16

**Figure 2:**
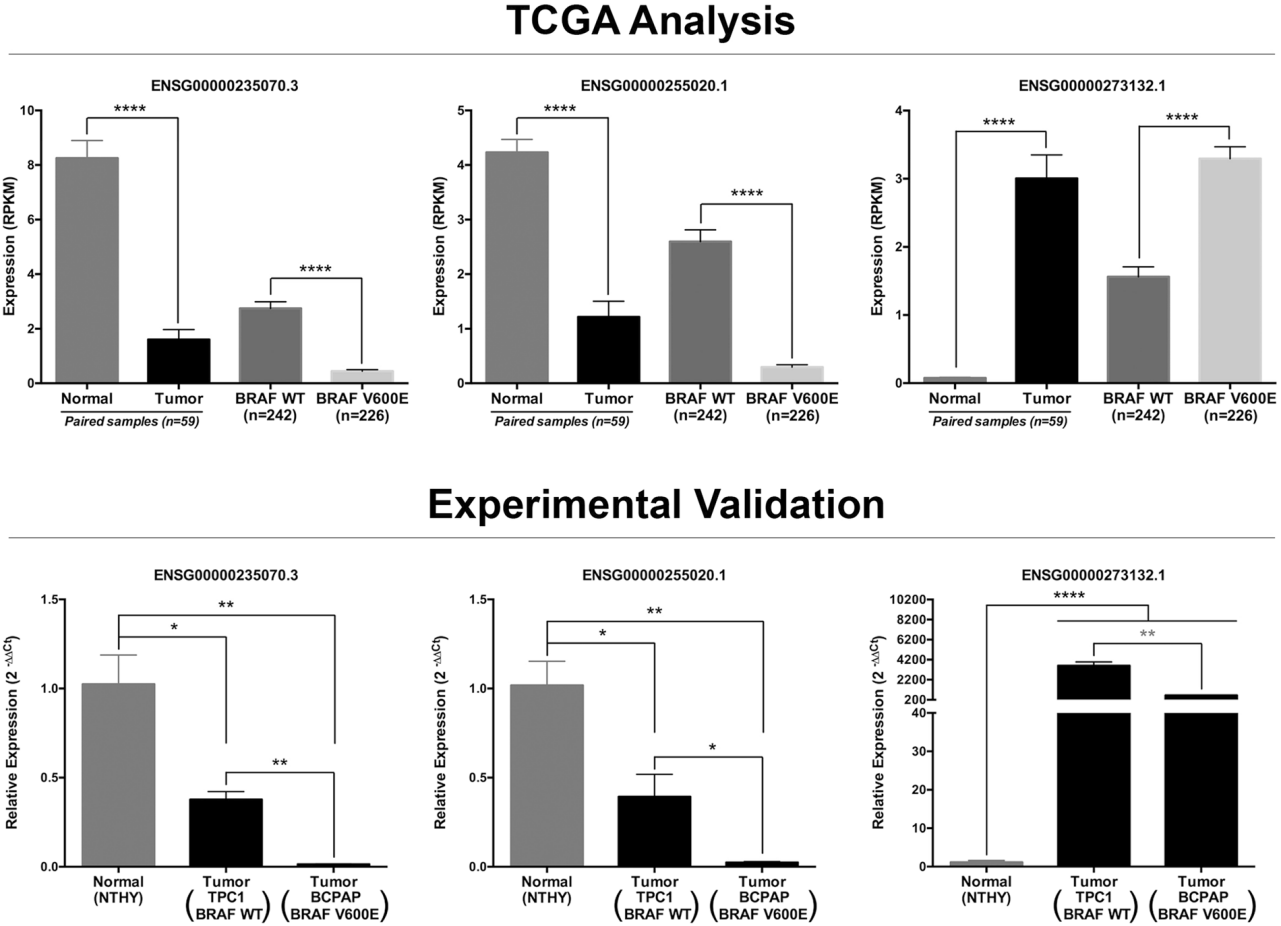
Experimental validation of DE lncRNAs. Upper part of panel displays the expression levels of the indicated lncRNAs in the TCGA analyses. The nonparametric Mann–Whitney test was applied due to the non-Gaussian expression distribution and p-value was assigned. Lower part of panel displays the experimental validation of these lncRNAs measured by qRT-PCR and calculated with 2^−ΔΔCt^ method using RPL19 (Ribosomal Protein L19) as endogenous control. Experiments with three biological replicates were performed using two technical replicates for each sample. These results are representative of at least two independent experiments. Values are plotted as expression mean ± Standard Error of Mean (SEM). Unpaired two-tailed t-Test assigned the p-value.

Experimental validation using qRT-PCR was performed to demonstrate the reliability of the bioinformatics analyses applied. From the top 25 positively DE lncRNAs and from the top 20 negatively DE lncRNAs, it were selected for validation those lncRNAs with low adjusted p-values to minimize expression variability, especially in the comparison BRAF^WT^ × BRAF^V600E^ tumor, among others characteristics (for detailed information see Methods). From a total of 5 DE lncRNAs selected for validation from the TCGA analysis (Fig. 2 and Supplemental Fig. S1, upper part), 4 DE lncRNAs were validated using thyroid cell lines (Fig. 2 and Supplemental Fig. S1, lower part), which strengths the reliability of this bioinformatics analysis, although the experimentally tested set of lncRNAs constitutes a relatively small sampling. Considering all comparisons, we obtained a very expressive validation efficiency [from a total of 8 different comparisons (Normal × Tumor and BRAF^WT^ × BRAF^V600E^), 6 were validated]. Downregulation of *ENSG00000235070.3* and *ENSG00000255020.1* in PTC was confirmed in the tumor cell lines TPC1 (BRAF^WT^) and BCPAP (BRAF^V600E^) compared to the normal immortalized cell line NTHY (Fig. 2). Also, downregulation of their expression was in accordance to the bioinformatics analysis, since lower expression for both of them was observed in BCPAP (BRAF^V600E^) compared to TPC1 (BRAF wild type) (Fig. 2). Noteworthy, is that due to the very low abundance of *ENSG00000255020.1* in the BCPAP cell line, qRT-PCR resulted in two unspecific melting peaks, which did not influenced the results. Upregulation of *ENSG00000273132.1* in PTC was confirmed, however its overexpression in BRAF^V600E^ tumors was not observed in the cell line BCPAP compared to TPC1 (Fig. 2), maybe due to the small log2 fold change value (1.69) of this comparison. Overexpression of *ENSG00000230498.1* in BRAF^V600E^ PTC compared to BRAF wild type tumors was also confirmed (Supplemental Fig. S1); nevertheless ENSG00000247311.2 was undetectable in both TPC1 and BCPAP cells (Supplemental Fig. S1).

### Clustering lncRNAs identifies two groups with similar expression patterns

For downstream analyses, we increased the stringency of differentially expressed lncRNAs between Normal × Tumor (log2 fold change > 3 or < −3; adj. p-value <1 × 10^−7^, n = 73; Fig. 1D) and between WT × BRAF^V600E^ (log2 fold change >2.5 or < −2.5; adj. p-value < 1 × 10^−4^, n = 59; Fig. 1E) to analyze the lncRNAs that were most DE. Hierarchical clustering was used to organize patients or lncRNAs into groups according to the expression levels of DE lncRNAs.

Results demonstrated that this set of lncRNAs is capable of clustering, majorly, normal and cancer patients in two distinct groups (Supplemental Fig. S2A). Clustering lncRNAs by Spearman correlation among all DE lncRNAs also identified two groups of lncRNAs highly positively correlated or negatively correlated (Supplemental Fig. S3A).

Hierarchical clustering was also performed with a more stringent set of DE lncRNAs between WT and BRAF^V600E^, which allowed the clustering of two groups enriched with WT and BRAF^V600E^ patients, respectively (Supplemental Fig. S2B). Clustering lncRNAs by Spearman correlation among all DE lncRNAs also identified two groups highly positively correlated or negatively correlated lncRNAs (Supplemental Fig. S3B).

### Indirectly validated lncRNAs’ targets are involved in several oncogenic processes

As almost the totality of the identified DE lncRNAs in both conditions (Normal × Tumor and WT × BRAF^V600E^) is uncharacterized, we used prediction methods to identify a possible interaction between lncRNAs and mRNAs/microRNAs. Predicted mRNAs and microRNAs (targets of DE lncRNAs) were compared to differentially expressed mRNAs and microRNAs (log2 fold change >1 or <−1; adj. p-value < 0.05) calculated from the same TCGA patients. Predicted mRNAs/microRNAs that were also identified as DE were considered as indirectly validated targets.

**Figure 3:**
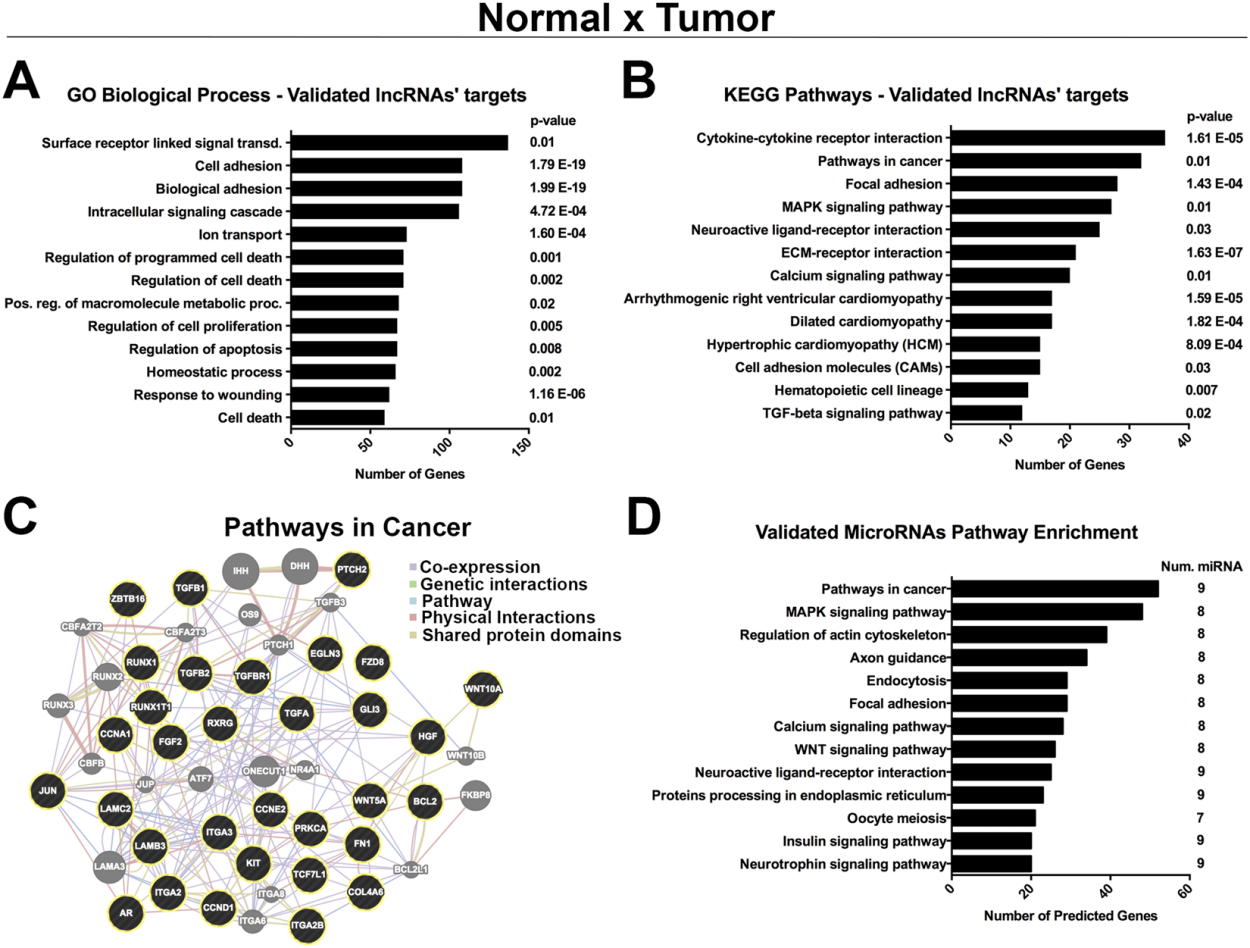
Indirectly validated targets of the DE lncRNAs between N × T are involved in cancer-related processes. (**A**) GO biological processes and (**B**) KEGG enriched pathways of the indirectly validated mRNA targets of the DE lncRNAs between N × T. (**C**) Proteins’ network of genes linked to the pathways in cancer, where black circles are validated targets and grey circles are connective proteins. (**D**) KEGG Pathways enrichment of the indirectly validated microRNAs targets of the DE lncRNAs between N × T.

A total of 1109 DE mRNAs (Table 2 and Supplemental Table S8) and 26 DE microRNAs (Supplemental Table S9) were found to be predicted targets of the DE lncRNAs between Normal and Tumor samples and were considered as indirectly validated targets. Gene ontology and KEGG pathways enrichment of these validated mRNAs demonstrated that most of the genes are involved in surface receptors responsible for signal transduction and cell adhesion, as well as, regulation of cell death, proliferation and apoptosis (Fig. 3A). Enriched pathways (Fig. 3B) were composed of cytokine-cytokine receptor interaction, pathways in cancer (Fig. 3C), focal adhesion, MAPK pathway and calcium signaling pathway. Validated microRNAs were also used to determine enriched pathways based on their predicted targets calculated elsewhere (Fig. 3D). Genes involved in cancer and MAPK pathways were the most enriched pathways. Interestingly, some genes predicted to be targets of the validated microRNAs were also DE expressed in our analysis (Supplemental Table S2), such as upregulation of the MAPK constituents, CACNG4, CACNA1E, DUSP4, TGFBR1, FGF1, FGF2 and MAP3K1. Enriched pathways were extended to genes involved in cancer, focal adhesion and calcium signaling (Fig. 3D).

**Table 2:** Top 5 upregulated and 5 downregulated DE lncRNAs between paired Normal × Tumor with examples of indirectly validated targets.

Ensembl	log2 FC	Adj. p-value	Upregulated indirectly validated targets	Downregulated indirectly validated targets
	N × T	N × T		
*ENSG00000223914.1*	7.04	8.69E-11	VGLL1, GDF6, FAM19A2, HRH1	RPS6KA5, RNF150, ANK2, FOSB
*ENSG00000250748.2*	7.01	4.56E-09	FUT3, GPR115, CAPN8, COL7A1	SVEP1, LMOD1, DPT, KCNA1
*ENSG00000214797.3*	7.01	6.39E-09	TMEM130, HES2, KCP, DTX4	CDHR3, PAK3, RASSF6, NWD1
*ENSG00000273132.1*	5.49	4.17E-10	KLK6, ELFN2, C19orf59, SHISA6	SRF, CPXM1, LAYN, FAM163A
*ENSG00000230918.1*	5.49	1.17E-10	GRM4, DPP4, LRP4, SHISA6	EGR1, TFCP2L1, FOXJ1, ABCA9
*ENSG00000253288.1*	−7.43	1.03E-09	SLC6A20, KLK10, FUT3, HRH1	HAP1, SH2D6, FOXP2, ADH1B
*ENSG00000272479.1*	−7.19	1.16E-09	DMBX1, TMPRSS6, TMPRSS4, PPP1R1B	CUX2, PAX1, CLCNKB, FOSB
*ENSG00000261185.1*	−6.42	1.13E-10	B3GNT3, ELFN2, LRP4, SHISA6	NR4A1, C1QTNF7, RNF150, FAM180B
*ENSG00000254489.1*	−6.37	7.98E-15	SYTL5, HPCAL4, KCNQ3, CPNE4	RBM24, PGA3, GFRA1, RNF150
*ENSG00000260412.1*	−6.33	7.11E-09	CLDN16, PDE4C, LRG1, SHROOM4	SLC26A4, CDHR3, PAK3, NWD1

**Figure 4:**
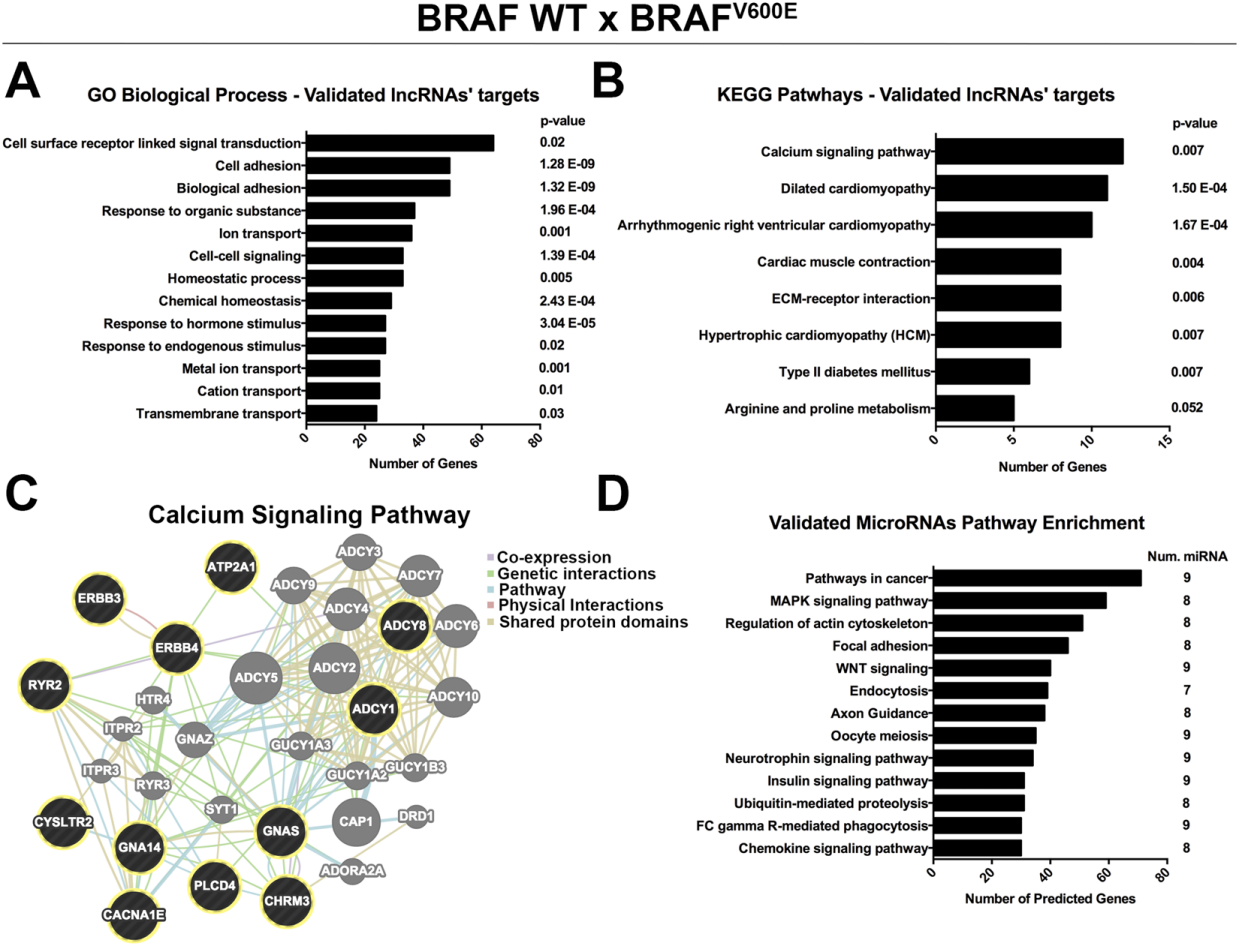
Indirectly validated targets of the DE lncRNAs between WT × BRAF^V600E^ are involved in oncogenic pathways. (**A**) GO biological processes and (**B**) KEGG enriched pathways of the indirectly validated mRNA targets of the DE lncRNAs between WT × BRAF^V600E^. (**C**) Proteins’ network of genes linked to calcium signaling pathway, where black circles are validated targets and grey circles are connective proteins. (**D**) KEGG pathways enrichment of the indirectly validated microRNAs targets of the DE lncRNAs between WT × BRAF^V600E^.

Between WT and BRAF^V600E^, 471 DE mRNAs (Table 3 and Supplemental Table S10) and 11 DE microRNAs (Supplemental Table S11) were indirectly validated. Gene ontology of these mRNAs demonstrated that most of the genes are also related to surface receptors involved in signal transduction and cell adhesion, but, additionally, with response to hormone stimulus and transmembrane transport (Fig. 4A). Enriched pathways (Fig. 4B) were constituted of calcium signaling pathway (Fig. 4C), cardiomyopathies and ECM-receptor interaction. KEGG enrichment pathway analysis of the validated DE microRNAs demonstrated participation of the MAPK and WNT pathways, as well as regulation of actin cytoskeleton and focal adhesion (Fig. 4D). Several pro-oncogenic genes were found to be upregulated in our analysis and were described as predicted targets of the validated DE microRNAs, as the example of MET and TGFBR1 genes (Supplemental Table S5).

**Table 3:** Top 5 upregulated and 5 downregulated DE lncRNAs between WT × BRAF^V600E^ with examples of indirectly validated targets.

Ensembl	log2 FC	Adj. p-value	Upregulated indirectly validated targets	Downregulated indirectly validated targets
	WT × BRAF V600E	WT × BRAF V600E		
*ENSG00000255595.1*	9.87	5.68E-05	TCAP, ITGA2, LY6G6C, BEND6	SLC5A5, ASTN1, PART1, IRX6
*ENSG00000214797.3*	5.19	4.92E-12	HES2, DTX4, KCP, LDLR	RNF157, HAP1, NWD1, SLC14A2
*ENSG00000260328.1*	4.04	2.26E-16	TMPRSS4	PRND, TMPRSS3, PREX2, CNTNAP2
*ENSG00000230498.1*	3.95	5.74E-14	C1orf106, ADAMTS14, DMBX1, DUSP13	FCGBP, GCGR, SOX3, PPP2R2C
*ENSG00000256916.1*	3.62	3.51E-09	ELFN2, SPTBN2, MUC16, ZNF469	SSPO, ASXL3, CTNND2, CNTNAP2
*ENSG00000267674.1*	−8.55	3.89E-13	VSIG1, LDLR	ADM2, ST3GAL6, NWD1, SLC5A8
*ENSG00000237396.1*	−6.35	3.05E-08	SIGLEC6, SDK1, C1QL2, MUC21	TFCP2L1, SCUBE1, SBSN, PAX1
*ENSG00000227947.1*	−6.22	5.72E-07	ELFN2, EPHA10, SLC30A3, SYT1	SFTPC, GATA5, SFRP1, MATN1
*ENSG00000224568.1*	−4.23	8.95E-18	SLC6A14, C1orf106	HIF3A, SYT13, SLC29A4, ARSF
*ENSG00000267214.1*	−4.18	1.17E-06	ELFN2, COL7A1, B4GALNT3	FAM124A, SULT1A2, SLC29A4, CNTNAP2

## Discussion

Long noncoding RNAs are arising as key participants in cancer establishment and progression by several oncogenic mechanisms^30, 32^. On the other hand, it is of urge interest the determination of how these lncRNAs are activated and how they can be associated with specific events or genotypes, such as point mutations. BRAF^V600E^ is the driver oncogenic mechanism with the greatest incidence in PTC^7^ and, therefore, any event correlated with this mutation will be necessary to understand BRAF^V600E^-induced aggressiveness.

This is the first study to identify DE lncRNAs correlated with BRAF^V600E^ in PTC and, besides that, we extended the cohort of paired normal and PTC samples to more accurately determine DE lncRNAs between these conditions.

**Figure 5:**
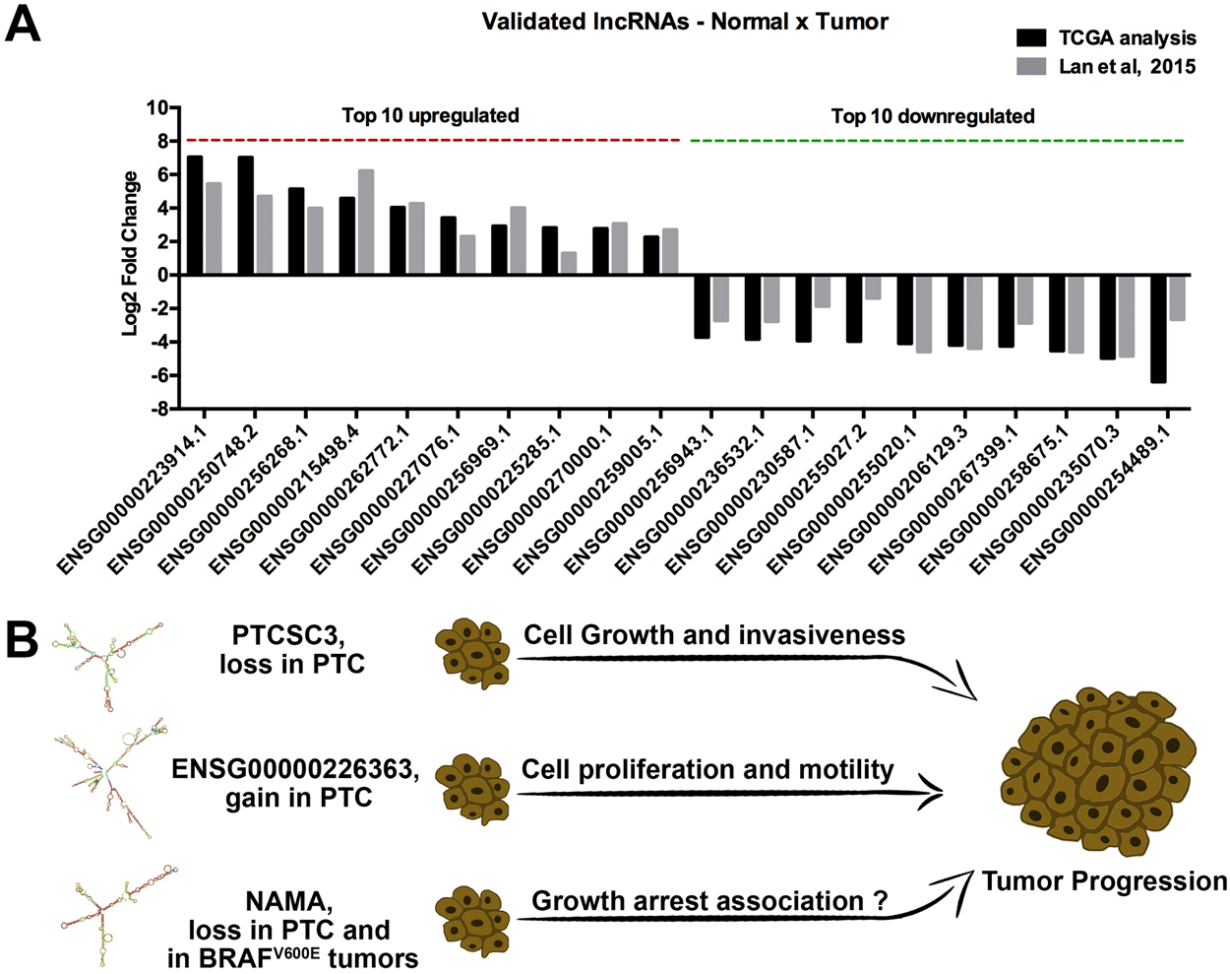
Experimentally validated lncRNAs are important to tumor malignancy. (**A**) Examples of DE lncRNAs between Normal and PTC, which were confirmed in a validation set^36^. (**B**) Differentially expressed lncRNAs in PTC that alter tumor malignancy.

We have identified 455 DE lncRNAs between paired normal and PTC samples. A total of 76 (log2 fold change >1 or < −1; adj. p-value < 1 × 10^−7^) lncRNA were previously reported as DE in thyroid cancer compared to adjacent normal thyroid^36^ (Fig. 5A and Supplemental Table S12). This validation set, together with the lncRNAs confirmed by experimental approaches (Fig. 2 and Supplemental Fig. S1), confers consistency to our analysis. Additionally, a diversity of DE lncRNAs identified in our analysis were reported in individual studies as altered in PTC samples, such as *ENSG00000259104.2* ^38, 39^, *ENSG00000236130* ^40^, *ENSG00000226363* ^37^, *ENSG00000271086* ^39, 41^, *ENSG00000223914* ^37^ and *ENSG00000187979* ^37^.

*ENSG00000259104.2* (PTCSC3), which is downregulated in the tumor samples (log2 fold change −1.40; adj. p-value 1.11E-12) was previously reported as having thyroid-specific expression and decreased expression in PTC^38, 39^. Interestingly, the risk allele [T] associated with SNP rs944289, located at PTCSC3’s promoter, affects the binding site of C/EBPα and C/EBPβ (PTCSC3 activators), reducing its expression. Restoration of PTCSC3 expression in PTC cells inhibited cell growth and affected the expression of genes involved in DNA replication/repair, cellular movement and cell death^38^. Also PTCSC3 ectopic expression reduces cell proliferation and increases cell cycle arrest and apoptosis^39^, while reducing cell motility and invasiveness through S100A4 downregulation^42^ (Fig. 5B).

*ENSG00000236130* (PTCSC2), was also reported as having decreased expression in PTC^40^, which was confirmed in our analysis (N × T log2 fold change −1.03; adj. p-value 3.12E-09). The risk allele [A] of rs965513 was significantly associated with low expression of unspliced PTCSC2 in unaffected thyroid tissue, however this correlation was not extended to PTC samples^40^.

*ENST00000426615* (*ENSG00000226363*) is another lncRNA that we identified as upregulated in PTC (NxT log2 fold change 3.87; adj. p-value 2.36E-05), which was experimentally demonstrated to be overexpressed in this cancer, inducing cell proliferation and motility^37^ (Fig. 5B).

Our analysis also confirmed the differential expression (N × T: log2 fold change −2.42; adj. p-value 3.96E-11) of the previously reported lncRNA *ENSG00000271086* (NAMA), which is downregulated in PTC compared to normal tissues^39, 41^ and in BRAF^V600E^ tumors compared to wild type tumors^39^ (Fig. 5B). NAMA is induced by inhibition of the MAPK pathway, growth arrest and DNA damage^41^ and our analysis also demonstrated that NAMA is downregulated in BRAF^V600E^ patients (WT × BRAF^V600E^ log2 fold change −1.66; adj. p-value 2.02E-15). All these independently validated lncRNAs demonstrate the reliability of our study (Fig. 5).

Similarity matrix based on Spearman correlation identified clusters of DE lncRNAs between Normal × Tumor (Supplemental Fig. S3A) and WT × BRAF^V600E^ (Supplemental Fig. S3B) with similar expression patterns, which can provide evidence for further studies to determine common upstream regulators.

Indirectly validated targets of the DE lncRNAs between Normal × Tumor are involved in a diversity of biological processes (Fig. 3A). For instance, it was noticed an overrepresentation of adhesion molecules, such as downregulation of CDH16, which was already reported as a potential marker for PTC^43^. Along with CDH16, many other cadherins were identified as validated targets of DE lncRNAs, such as CDH2, CDH3, CDH4, CDH6, CDH11 and CDH24 (Supplemental Table S8). Another highly enriched biological process was the regulation of programmed cell death (Fig. 3A), represented by the upregulation of the antiapoptotic SOX4^44^ and TP63^45^ in PTC samples. Enriched pathways (Fig. 3B) as cytokine-cytokine receptor interaction, focal adhesion and MAPK pathways were already reported in the first study of DE lncRNA with paired Normal × PTC samples^36^, providing further support for future research. It was observed an enrichment of MAPK-related genes, represented in our results by upregulation, in the tumor samples, of TGB1, TGFB2 and TGFBR1 that were shown to activate the MAPK pathway^46^. Interestingly, pathway enrichment analysis of indirectly validated microRNAs (Fig. 3D) demonstrated a convergent tendency to genes involved in cancer, MAPK pathway and focal adhesion, which were also observed with the validated mRNAs.

Indirectly validated targets of the DE lncRNAs between WT × BRAF^V600E^ tumors were demonstrated to be involved with cell surface receptors responsible for signal transduction and with cell adhesion (Fig. 4A). Pathway enrichment analysis, identified genes involved in calcium signaling and ECM-receptor interaction, which were already reported as an early transcriptome change in BRAF^V600E^-associated mouse model^24^. Interestingly, we also observed several genes correlated with cardiomyopathies that are mostly related to calcium regulation in cardiac muscle cells (Fig. 4B). Calcium (Fig. 4C) and MAPK cascade (represented by BRAF^V600E^ group) are tightly involved, where calcium modulates the protein interaction properties of ERKs, affecting the subcellular localization and influencing the distribution of their targets^47^. Calcium can also stimulates MEK through Ras activation^48^. Therefore, these results can support a future investigation to answer if BRAF^V600E^-stimulated MAPK activation can be reinforced by calcium modulation induced by the DE lncRNAs. Additionally, MAPK stimulation may be supported by the indirectly validated microRNAs, since pathway analysis demonstrated an enrichment of microRNAs’ targets in cancer and MAPK pathways (Fig. 4D). Interestingly, predicted targets of the DE microRNAs were also differentially expressed in our analysis, such as the upregulation of TGFBR1 and downregulation of PRKACB (Supplemental Table S5).

Concluding, our extended cohort of paired Normal and PTC patients identified new DE lncRNAs and confirmed many other lncRNAs already reported. Additionally, to our knowledge, this is the first study to identify BRAF^V600E^-correlated lncRNAs in PTC, which will provide support for future studies aiming to identify BRAF^V600E^-linked events in attempt to optimize therapeutic treatment and diagnosis/prognosis of this aggressive PTC genotype.

## Methods

### Data analysis

Thyroid Carcinoma (THCA) clinical information, mRNA and microRNA data expression data were downloaded from The Cancer Genome Atlas (TCGA) online platform (https://tcga-data.nci.nih.gov/tcga/), as January 2016. Mutations data were retrieved through cBioPortal^49^. LncRNA RPKM expression levels corresponding to TCGA patients were downloaded through TANRIC^50^, which obtained the genomic coordinates of 13,870 human lncRNAs from the GENCODE Resource (version 19)^51^ and further filtered out those lncRNA exons that overlapped with any known coding genes based on the gene annotations of GENCODE and RefGene, resulting in 12,727 lncRNAs^50^.

BRAF^V600E^ patients were selected to form the BRAF^V600E^ group (n = 226), which excluded any other type of BRAF mutations. Wild Type group (n = 242) was formed by patients without any somatic mutation in BRAF gene, but patients with mutations in HRAS, NRAS, KRAS, EIF1AX, PPM1D, RET and NTRK1 were considered. It were selected only the patients with papillary thyroid cancer diagnosis.

### Differential Expression Analysis

For differential expression analysis of mRNA and microRNA was used edgeR package^52^ through TCGAbiolinks^53^. To identify differentially expressed lncRNAs between groups, it was used the paired/unpaired Student t test to assess the statistical difference of mean expression values between the two groups^50^. LncRNA with median value equal zero were excluded and fold change was calculated using median expression values. In all differential analysis, p-values were adjusted for False Discovery Rate (FDR) < 0.05 as multiple hypothesis test correction method.

### RNA extraction, Reverse-transcription and qPCR

Total RNA was phenol-chloroform extracted from cell lines Nthy-ori3-1 (NTHY–immortalized human thyroid follicular epithelial cell), TPC1 (papillary thyroid carcinoma- RET/PTC1 rearrangement) and BCPAP (papillary thyroid carcinoma–BRAF^V600E^) using TRIzol reagent (Invitrogen) according to the manufacter’s instructions. Four µg of total RNA was reverse transcribed using M-MLV Reverse Transcriptase (Invitrogen) in the presence of 100 ng of random hexamers primers according to the manufacter’s protocol. qPCR reaction was performed using 100 ng of cDNA, 1X Power SYBR Green PCR Master Mix (Applied Biosystems) and specific primers. Amplification and detection were performed using ViiA7 TM Real-Time PCR System (Applied Biosystems). Relative gene expression was calculated using the QGENE program and calculated with 2^−ΔΔCt^ method using RPL19 (Ribosomal Protein L19) as endogenous control.

Primers used (5′-3′): RPL19 (Fw-TCTCATGGAACACATCCACAA; Rv-TGGTCAGCCAGGAGCTTCTT), *ENSG00000273132.1* (Fw-CTAGCTGCCAGCAGTGACAA; Rv-GCGAGAGCACAGATGACCAC), *ENSG00000230498.1* (Fw-CCCTGGGTGATGAAGATGAG; Rv-TGGGATCCCTTTTTTGTCCG), *ENSG00000235070.3* (Fw-TGACTCCAAGTTCACGCAGC; Rv-GTGGATGAGTTGTGTGCTGG), *ENSG00000255020.1* (Fw-AGTGACGTGGGGAAGAAACG; Rv-CGACATATTTCAAGGGCGCC) and ENSG00000247311.2 (Fw-GCTGTGAGTGACTCTTCAGC and ACAGACACACCCAGGAACAA).

To select the above DE lncRNAs for validation, it was taken in consideration one of the major characteristics of lncRNA, that is, high heterogeneous expression across the same tumor and even the same cell line. Long noncoding RNA’s expression is tightly regulated by a wild range of cellular responses and, due to the markedly lower transcriptional levels of lncRNAs, the expression variability inside the same group of patients is expected. Taking this aspects in consideration, from the top 25 positively DE lncRNAs and from the top 20 negatively DE lncRNAs, it were selected for validation those lncRNAs with low adjusted p-values to avoid this variability, especially in the comparison BRAF^WT^ × BRAF^V600E^ tumor, which is the focus of this research. As another desirable characteristic, most lncRNAs selected, presented at least in one group (normal thyroid, PTC, BRAF^WT^ or BRAF^V600E^) a median expression greater than 1 RPKM (reads per kilo base per million mapped reads). Added to that, it was given preference for those lncRNAs without isoforms (seen that many lncRNAs have annotation errors) and that present at least 2 exons, which are more stable and would allow the PCR primers to be located in different exons.

### Prediction of lncRNAs targets

To identify the possible target genes of the selected (Fig. 1D and E) differentially expressed lncRNAs via cis- or trans-regulatory effects, two previously described approaches were used^36, 54^. The genes transcribed within a 10 kb window upstream or downstream of lncRNAs were considered as cis-target genes^36, 54^. The second method was used to identify trans-targets and is based on mRNA and microRNA sequence complementarity with the query lncRNA. For mRNA interactions we used a pre-computed database that catalogs the predicted lncRNA–RNA interactions^55^, where the accessible regions of the query lncRNA and possible targets (mRNA/lncRNA) are extracted, the binding energies of pairs of sequences (target and query) around the seed matches are evaluated and the minimum interaction energy of the joint secondary structures is calculated^55^. The 500 predicted targets (mostly constituting repeated targets with different interaction sites) with the lowest minimum free energies under −20 kcal/mol were taken in consideration for downstream analysis. For lncRNA and microRNA interaction prediction it was used *rna22*, a method for identifying microRNA-binding sites and their corresponding heteroduplexes^56^. It were selected those microRNAs with a folding energy lower than −20 kcal/mol.

### Indirect Validation

As interaction prediction methods are susceptible to error and to minimize this, we compared the predicted targets of the differentially expressed lncRNAs with the differentially expressed mRNAs and microRNAs^36^ calculated with the TCGA patients, because we consider that the targets of DE lncRNAs would possibly be DE in TCGA analysis. With this approach, we intended to enrich our analysis for targets with a greater propensity to be occurring biologically.

### Gene ontology, pathway enrichment and protein-protein interaction network

Indirect validated targets of the DE lncRNAs were loaded into the Database for Annotation, Visualization and Integrated Discovery (DAVID)^57^, which returned the gene ontology of the query genes and identified enriched Kyoto Encyclopedia of Genes and Genomes (KEGG) pathways^58^. miRSystem^59^ was used to calculate enriched pathways based on the predicted targets of the query microRNAs, which in this case, were the DE microRNAs in both conditions (Normal × Tumor and WT × BRAF^V600E^). For protein-protein interaction network it was used Genemania^60^.

## Electronic supplementary material

Supplemental Figures

Supplemental datasets
