# Supplementary material for: Identification of Long Noncoding RNAs Deregulated in Papillary Thyroid Cancer and Correlated with BRAFV600E Mutation by Bioinformatics Integrative Analysis
Source: Sci Rep. 2017 May 10;7:1662. doi: 10.1038/s41598-017-01957-0 (PMC5431778; doi:10.1038/s41598-017-01957-0)
Supplement: Supplementary file 1 — Supplemental Figures [file 41598_2017_1957_MOESM1_ESM.pdf]

Identification of Long Noncoding RNAs Deregulated in Papillary Thyroid Cancer and Correlated with BRAF^{V600E} Mutation by Bioinformatics Integrative Analysis

Lucas Goedert[1,2,#]; Jessica Rodrigues Plaça[2,3,#]; Cesar Seigi Fuziwara[4]; Maiaro Cabral Rosa Machado[1]; Desirée Rodrigues Plaça[5]; Palloma Porto Almeida[6]; Talita Perez Sanches[1]; Jair Figueredo dos Santos[7]; Amanda Cristina Corveloni[8]; Illy Enne Gomes Pereira[1]; Marcela Motta de Castro[1]; Edna Teruko Kimura[4]; Wilson Araújo Silva Jr[2,9] ; Enilza Maria Espreafico[1,2].

1 – Department of Cell and Molecular Biology, Faculty of Medicine of Ribeirão Preto, University of São Paulo, Ribeirão Preto, São Paulo, Brazil.
2 – National Institute of Science and Technology in Stem Cell and Cell Therapy and Center for Cell-Based Therapy, Ribeirão Preto, São Paulo, Brazil.
3- Clinical Oncology, Stem Cell and Cell Therapy Program, Ribeirão Preto Medical School, Ribeirão Preto, São Paulo, Brazil.
4- Department of Cell and Developmental Biology, Institute of Biomedical Sciences, University of São Paulo, São Paulo, São Paulo, Brazil.
5 – Biotechnology Program, Federal University of São Paulo, São José dos Campos, São Paulo, Brazil.
6 - Biotechnology Program, Federal University of Bahia, Vitória da Conquista, Bahia, Brazil.
7 – Biomedicine Program, Federal University of Pernambuco, Recife, Pernambuco, Brazil.
8 – Biomedicine Program, State University of Londrina, Londrina, Paraná, Brazil.
9 - Department of Genetics, Ribeirão Preto Medical School, and Center for Integrative System Biology – CISBi-NAP/USP, University of São Paulo, Ribeirão Preto, São Paulo, Brazil.

# Authors with equal contribution

Corresponding author:
EME - emesprea@fmrp.usp.br

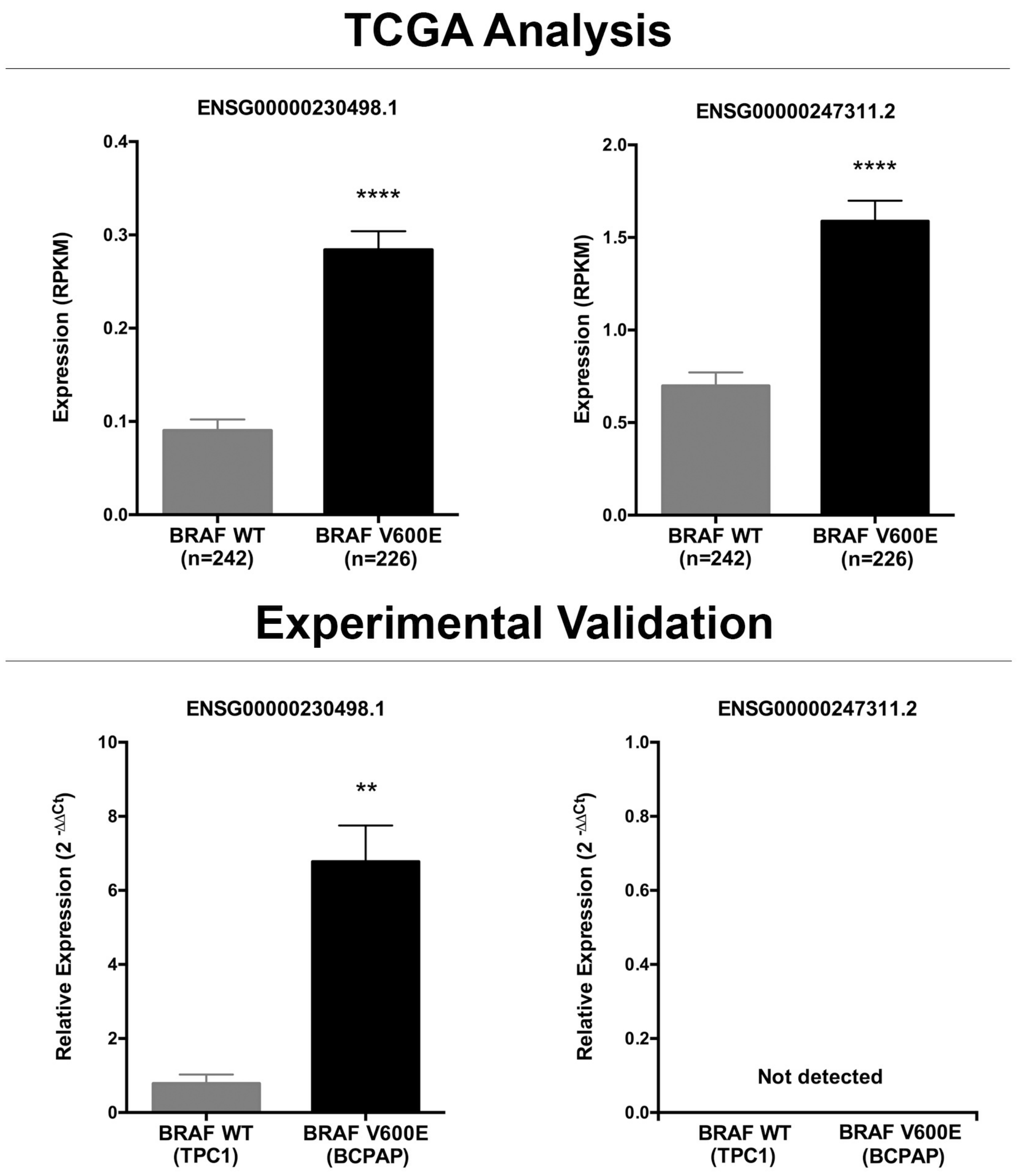

Supplementary Figure S1: **Experimental validation of DE lncRNAs.** Upper part of panel displays the expression levels of the indicated lncRNAs in the TCGA analyses. The nonparametric Mann–Whitney test was applied due to the non-Gaussian expression distribution and p-value was assigned. Lower part of panel displays the experimental validation of these lncRNAs measured by qRT-PCR and calculated with $2^{-\Delta\Delta Ct}$ method using RPL19 (Ribosomal Protein L19) as endogenous control. Experiments with three biological replicates were performed using two technical replicates for each sample. These results are representative of at least two independent experiments. Values are plotted as expression mean ± Standard Error of Mean (SEM). Unpaired two-tailed t-Test

assigned the p-value. For *ENSG00000247311.2* experimental validation, we also tried to perform RNA reverse transcription into cDNA using a combination of random hexamers and oligo-dT primers, however the expression continued undetectable.

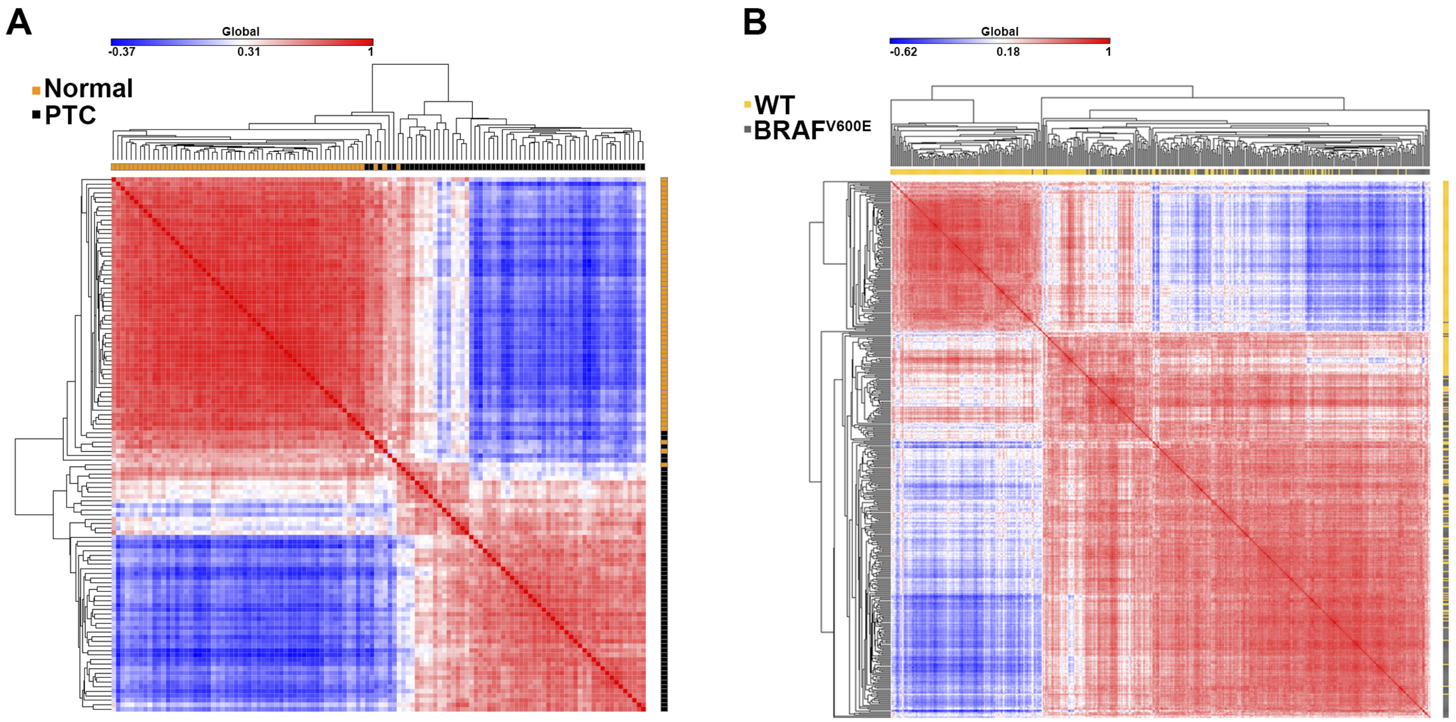

Supplementary Figure S2: **Expression of the DE lncRNAs differs between the comparison groups.** (**A**) N x T and (**B**) WT x BRAF^{V600E} similarity matrixes comparing patients according to the expression of all DE lncRNAs. For hierarchical clustering, Spearman rank correlation, average linkage was performed.

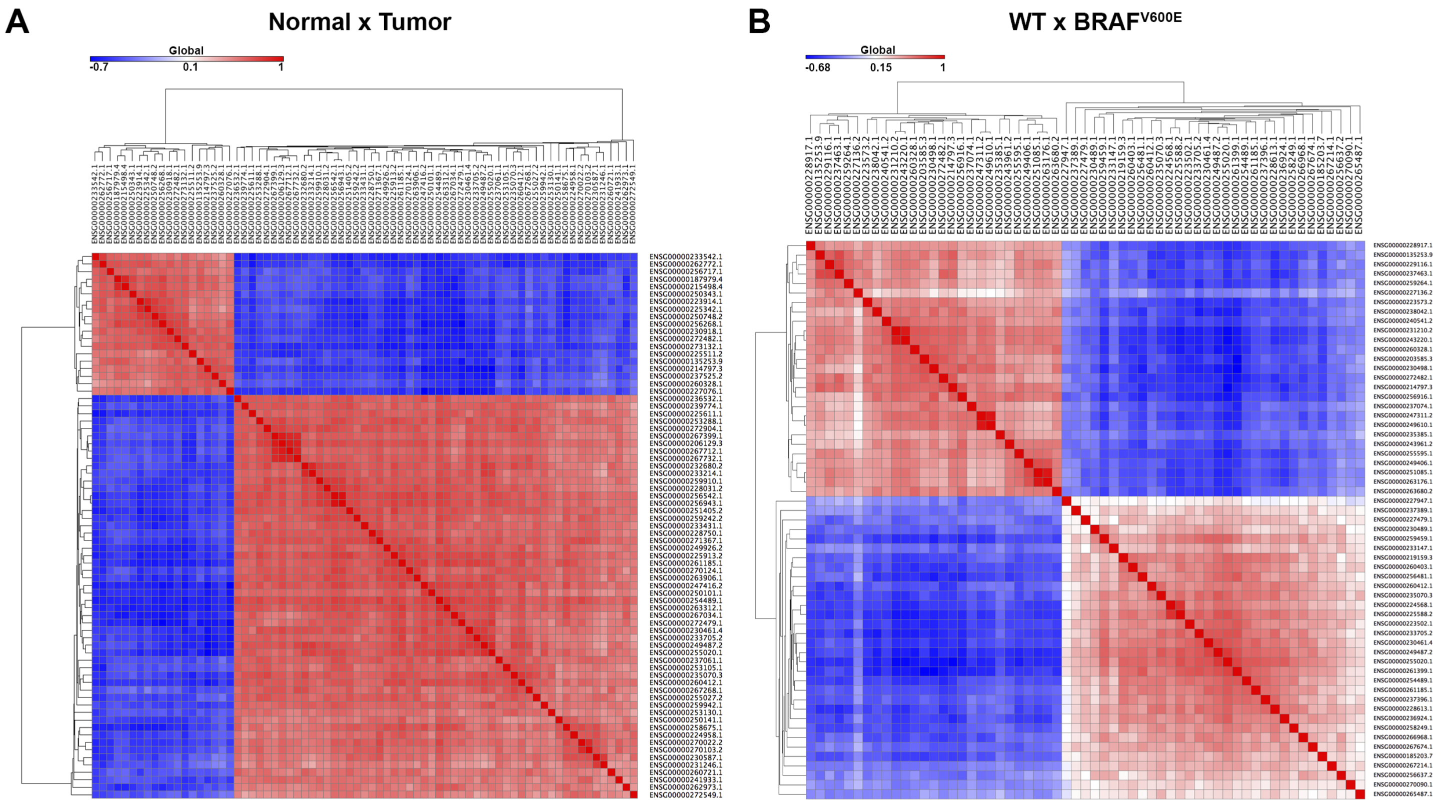

Supplementary Figure S3: **Correlated lncRNAs were identified**. (**A**) Similarity matrix* of DE lncRNAs between N x T (log2 fold change >3 or <-3; adj. p-value $<1x10^{-8}$). (**B**) Similarity matrix* of DE lncRNAs between WT x $BRAF^{V600E}$ (log2 fold change >2.5 or <-2.5; adj. p-value $<1x10^{-5}$). * For hierarchical clustering, Spearman correlation, average linkage was performed.
